# First burials give insights into mortuary rites at Göbeklitepe, Early Neolithic Türkiye

**DOI:** 10.1371/journal.pone.0358979

**Published:** 2026-09-22

**Authors:** Julia Gresky, Lee Clare

**Affiliations:** 1 Faculty of Biology and Psychology, Georg-August University, Göttingen, Germany; 2 Division of Natural Sciences, German Archaeological Institute, Berlin, Germany; 3 Istanbul Department, German Archaeological Institute, Istanbul, Türkiye; University of Szeged Institute of Biology: Szegedi Tudomanyegyetem Biologia Intezet, HUNGARY

## Abstract

Since the beginning of excavations at Göbeklitepe, a Pre-Pottery Neolithic (PPN) site in southeastern Türkiye, fragmented human bones have been recovered from the fill of the special (monumental) buildings. These fragments derive from males and females of all age categories and exhibit various taphonomic alterations. Compared with contemporaneous Anatolian burial customs, certain parallels exist; however, the seemingly random distribution of human bone fragments at Göbeklitepe remains enigmatic. Recently, the first two intact burials were discovered in Pre-Pottery Neolithic B (8,700–8,000 BCE) contexts. The taphonomic evidence from these burials could explain the presence of the dispersed human bone fragments. It appears that lower-lying parts of the site became inundated by slope deposits from higher areas of the mound, which contained human remains from originally intact burials. Nonetheless, this explanation does not rule out the possibility of additional mortuary practices, thereby underscoring the considerable diversity of burial rituals in Neolithic Anatolia.

## Introduction

**Fig 1:**
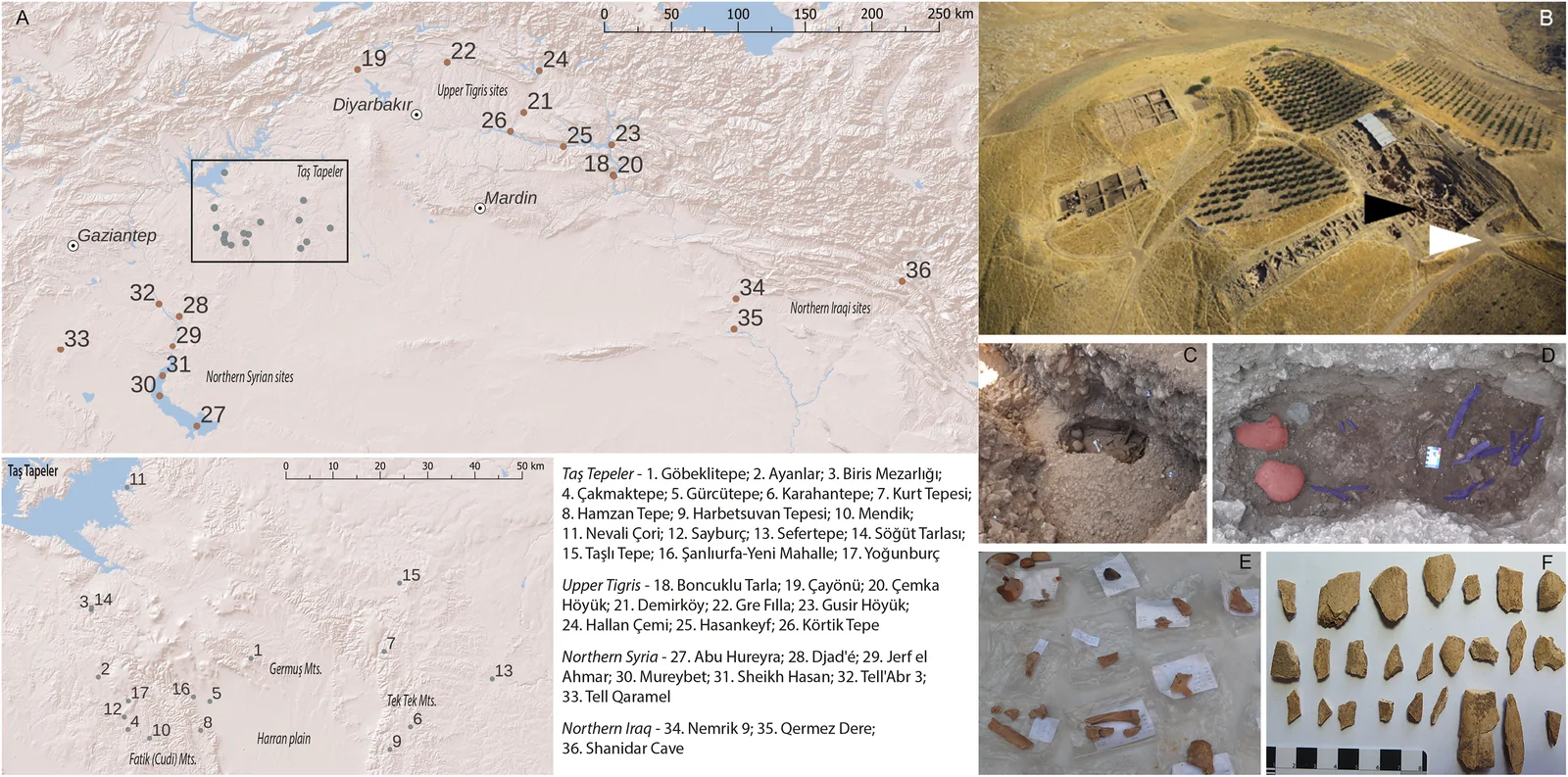
The site of Göbeklitepe with the location of the two burials and the fragmented human bones from the main excavation area. (A) Göbeklitepe in the Taş Tepeler region in Southeast Türkiye and contemporaneous sites; (B) Location of the two burials in trenches DR1 (white arrow) and L9-65 (black arrow) on the excavation area. E. Küçük, DAI, Göbeklitepe Research Project; (C) Collective burial in Drainage Channel 1 (DR1) beneath the floor of a rectangular Pre-Pottery Neolithic B building, C. Lelek-Tvetmarken, DAI, Göbeklitepe Research Project; (D) Two of the three cranial vaults (red) in the eastern corner of the burial, some of the long bones (blue) piled up in the western corner; (E) Collection of non-associated human bone fragments from the filling of the special buildings; (F) State of fragmentation of human bone fragments, J. Gresky, DAI.

Located 15 kilometres northeast of the city of Şanlıurfa in modern-day southeastern Türkiye, the archaeological site of Göbeklitepe is one of the most significant archaeological discoveries of the twentieth century (Fig 1A and 1B). Inscribed as a UNESCO World Heritage Site in 2018, Göbeklitepe has revealed evidence for early monumental architecture and a developed artistic and symbolic tradition, the latter reflecting prehistoric narratives and belief systems, primarily in the form of low and high reliefs of wild animals carved on its characteristic T-shaped pillars. As such, Göbeklitepe is a key site for understanding the transitional period from mobile hunter-foraging to sedentary farming in the upper Euphrates and Tigris basins, a core region of Neolithisation in Southwest Asia [1–3].

Since 2021, the Taş Tepeler Project, initiated by the Ministry of Culture of Türkiye, has broadened the research scope through excavations at several additional transitional Neolithic sites in the region, including Karahantepe, Sayburç, Sefertepe, Çakmaktepe, and Mendik, making the Şanlıurfa region one of the best-researched areas for this period [4,5].

The artificial settlement mound (tell) at Göbeklitepe accumulated over some 1,600 years, spanning the Pre-Pottery Neolithic A (PPNA; 9,600–8,700 BCE) to the Early/Middle Pre-Pottery Neolithic B (EPPNB/MPPNB, 8,700–8,000 BCE). While during the first two decades of research the site was interpreted as a ritual centre lacking significant permanent occupation [6], recent years have seen a reevaluation of this view, with increasing evidence for dwellings and domestic activities throughout its entire sequence [7–10]. The discovery of two subfloor human burials dating to the PPNB, presented in this contribution, provides yet another indication of the additional domestic function of the site.

Osteological analyses of the human bones from Göbeklitepe, initiated by JG in 2009, are ongoing. The assemblage (excluding the two burials) consists of highly fragmented human remains recovered from the excavated sediments within the special buildings and from the areas between them; consequently, the material is irregularly distributed across the entire site. To date, the assemblage comprises 928 individual, mostly unrelated fragments, characterised by a range of taphonomic alterations. In addition to natural processes, these alterations reflect intentional (human-mediated) treatments of the bones [11–15].

The origin of the human bones, particularly those retrieved from the fill of the special buildings, has been the subject of ongoing debate. This discussion centres on the nature of the fill material and the processes responsible for its accumulation. Earlier interpretations proposed that the buildings were intentionally (ritually) backfilled as part of their abandonment [16]. More recent studies, however, emphasise the role of erosion: the structures appear to have been inundated by deposits from higher-lying areas of the site via slope slides, caused by increased slope pressure from continuous building activities, possibly in combination with heavy rainfall and seismic events [7,9,17,18].

Based on the cumulative archaeological evidence, two potential origins for the human bones found within the fills of the special buildings can therefore be identified:

1) deliberate deposition of selected human bones as part of secondary or tertiary burial practices (e.g., the selection of skeletal areas, such as skulls and/ or long bones, or of a particular age group or sex, potentially reflected in the different treatments of bones).
2) burials from domestic contexts that became displaced and redeposited through erosion processes (e.g., presence of all skeletal parts from all age groups and sexes, with different treatments of the bones reflecting different burial customs).

Secondary burial customs are well known from Neolithic eastern Anatolia and are expressed in various forms [19–21]; however, the widespread dispersal and commingling of bone fragments observed in the fill of special buildings observed at Göbeklitepe has only one known parallel to date: At Karahantepe, approximately 70 human bone fragments, mainly from adults, have been collected from the fill of special buildings, adjacent dwellings and their surroundings; the majority of these are skull fragments that show signs of intentional modification, such as cutting, incising, and scraping, while others also show signs of exposure to fire [22].

Recently, the first two burials have been excavated at Göbeklitepe. The first burial was discovered in 2012 but excavated only in 2022, while the second was found and excavated in 2017 during construction works for a permanent protective shelter. These two burials, located close to the special buildings in the southeastern part of the mound, provide new insights into Pre-Pottery Neolithic burial customs at Göbeklitepe and its population. Moreover, through the study of these burials we aim to determine whether the bones from these features exhibit the same taphonomic alterations as those observed in the human bone assemblage recovered from the fill of the special buildings, and whether the dispersed fragments may derive from displaced burials in the surrounding area.

## Results

Archaeological context of the burial in trench DR1 (Fig 1C and 1D) and osteological information

This collective burial was discovered in 2017 during construction work for a drainage channel associated with the permanent protective shelter that now covers the Southeast-Hollow (main excavation area) of the mound. A kidney-shaped pit cut into the plaster floor of a rectangular PPNB building (Fig 1C) contained a collective burial comprising the skeletal elements of at least three relatively complete individuals (Fig 1C and 1D). Most of the skeletal elements were no longer in situ: the crania had been placed in the southeastern corner of the pit, postcranial elements were piled toward the centre, and some of the long bones were arranged along the western wall. The floor of the burial pit itself consisted of an additional plaster floor, likely belonging to an earlier building phase of the same structure.

**Fig 2:**
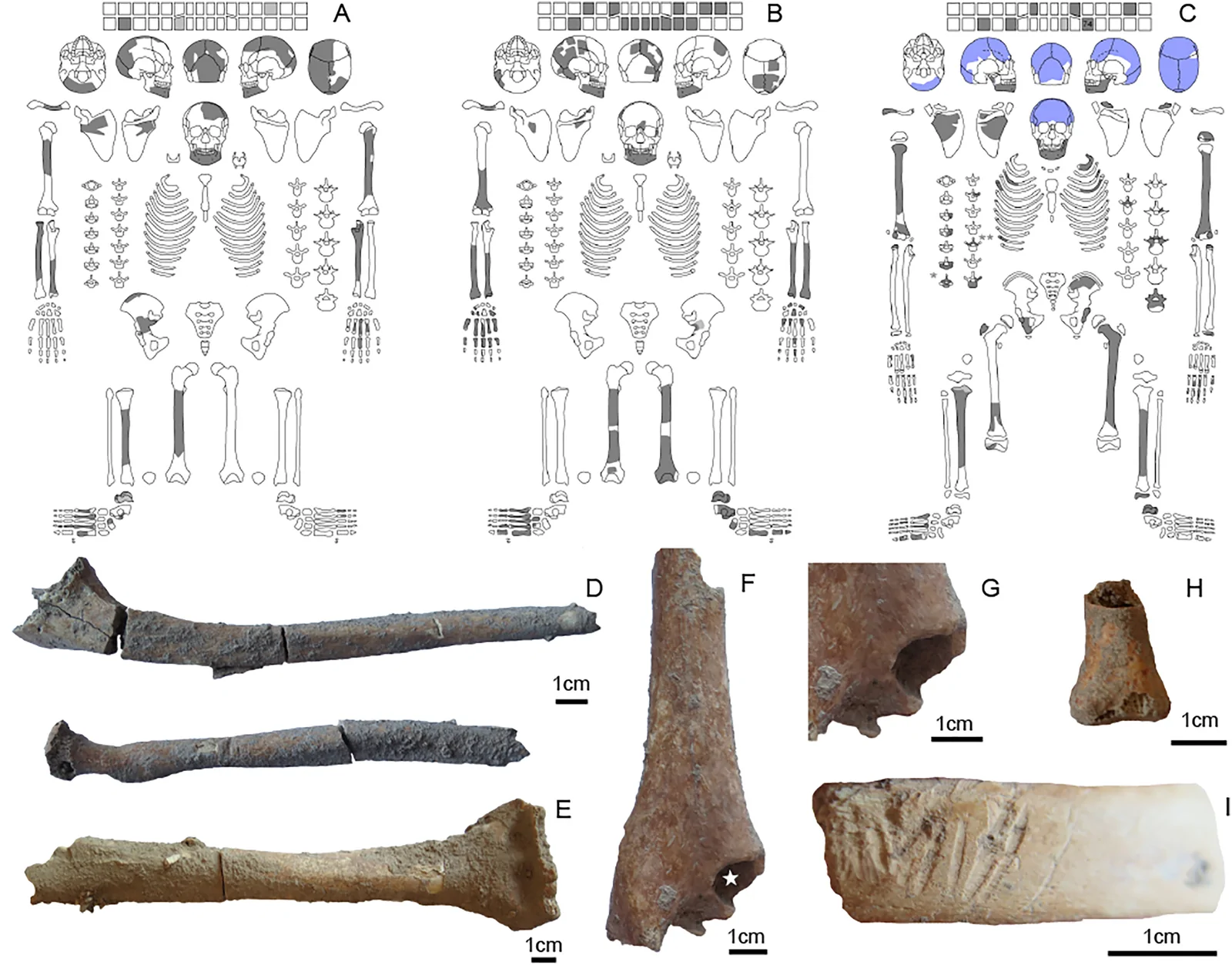
Individuals from the burial in trench DR1, showing taphonomic changes to the bones. Representation of bones of (A) skeleton 1, adult female; (B) skeleton 2, adult male; (C) skeleton 3, adolescent female. Dark grey indicates preservation of bone >75%, light grey marks bones with preservation <75%. Light blue is the cranium, which likely belongs to individual 3, but could also belong to a fourth, older individual. (D) Fragmentation of the left ulna and right radius of individual 1 with horizontal fractures; (E) Smooth fracture lines of a horizontal fracture of the left humerus of individual 2; (F, G) Gnawing marks at the lateral supracondylar ridge (star) of the right humerus of individual 2; (I) and on the outer surface of the ribs of individual 3; (H) Smooth fracture line of the third proximal phalanx of the left hand of individual 3. All images J. Gresky, DAI.

The presence of three crania and three mandibles, together with the absence of redundant skeletal elements, indicates a minimum of three individuals (Fig 2A–2C). The postcranial remains could be assigned accordingly: a female at least 35 years old (Individual 1, Fig 2A), a 20–30-year-old male (Individual 2, Fig 2B), and an 11–14-year-old female (for sex assessment, see supplementary information) (Individual 3, Fig 2C). However, a fourth individual may also be represented. One of the crania (Fig 2C, marked in blue) appears too mature to belong to the adolescent, and two metatarsal bones with unusually round shafts differ from those associated with the three primary individuals. In the three main individuals, nearly all anatomical regions are represented by at least a few bones (Fig 2A–2C).

### Preservation and taphonomic changes

The bone consistency ranges from dense to brittle, with the crania being particularly fragile and prone to breakage. The surfaces of most bone fragments are covered by a layer of calcareous deposits (61.4%, n = 116/189), affecting between 70–100% of their surface area. The left *radius* and *ulna* of Individual 3 are fixed in slight pronation due to these deposits, and several carpals and metacarpals of the right hand are fused by it. The bones are highly fragmented postmortem, with larger bones showing the highest fragmentation. The epiphyses of the long bones in the adult individuals are broken and missing (Fig 2D–2F), while the shafts survive only as larger fragments.

Most fractures exhibit the serrated fracture edges characteristic of postmortem breakage resulting from taphonomic forces such as soil pressure. However, several smooth fracture margins, helical breaks [23,24] indicating a certain amount of collagen still present in the bone, are present, the origin of which cannot be clearly classified as postmortem (Fig 2H, S1 (Table S1) in S1 File). Rodent gnawing marks are found primarily on bones belonging to Individual 2 (Fig 2F and 2G). In Individual 3, a series of parallel scratches on one rib (Fig 2I) may be of more recent origin, as indicated by their distinct colouring relative to the surrounding bone surface (S1 (Table S2) in S1 File). No cut marks, heat alterations, or traces of ochre application are visible on any of the bones.

**Fig 3:**
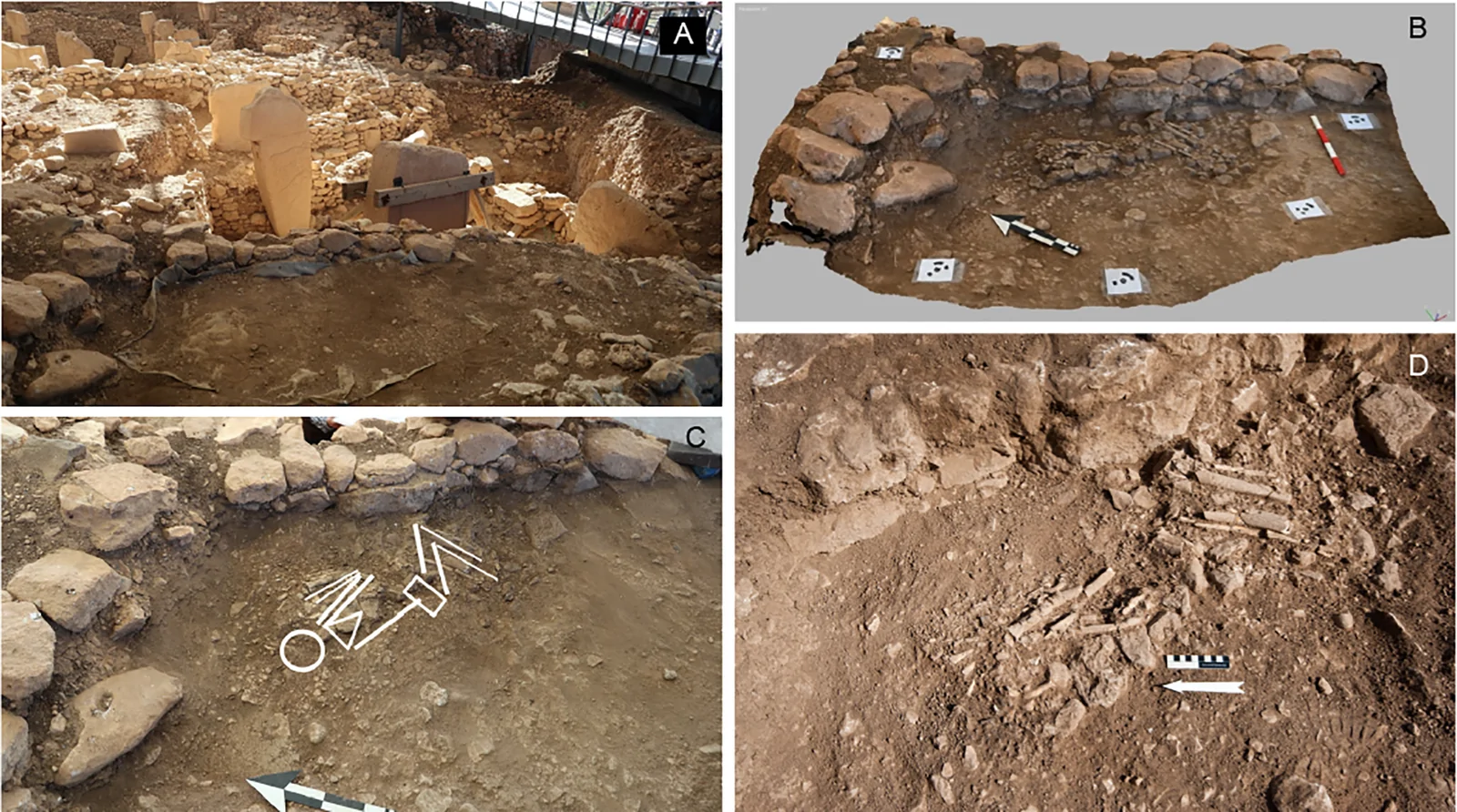
Individual from the burial in trench L09-65. (A) Location of the single burial in L09-65, adjacent to Special Building A, J. Gresky, DAI; (B) SFM (Structure-from-Motion) Orthophoto, June 2022, B. Waszk, DAI; (C) Single flexed interment (reconstructed with white lines) on its left side facing east, J. Gresky, DAI; (D) Bones of the skeleton at the start of the excavation in 2022, J. Gresky, DAI.

Archaeological context of the burial in trench L09-65 (Fig 3) and osteological information

This skeleton, excavated in 2022, was found against the eastern wall of a small space located immediately west of Special Building A. It had been placed in a flexed position on its left side, facing east (Fig 3C and 3D). Apart from the elements already excavated in 2012 and the displacement of the proximal foot phalanges, located near the midshafts of the right *tibia* and *fibula*, the skeleton remained in situ. It rested on a layer of fist-sized stones, without any clearly defined burial pit (Fig 3B). After the removal of the stone layer, additional bone fragments were recovered. Approximately 10 cm below the initial level, fragments of the left mandible, the right parietal bone in the area of the mastoid angle, and the left petrous portion were found near the cranium. Around 20 cm deeper, fragments of the right *ulna* and right *fibula* were uncovered in the region of the thorax. The sediment beneath the skeleton was very dense, composed of small to large stones, patches of plaster floor, small flint fragments, and animal bones. There was no evidence of animal activity, such as animal burrows.

**Fig 4:**
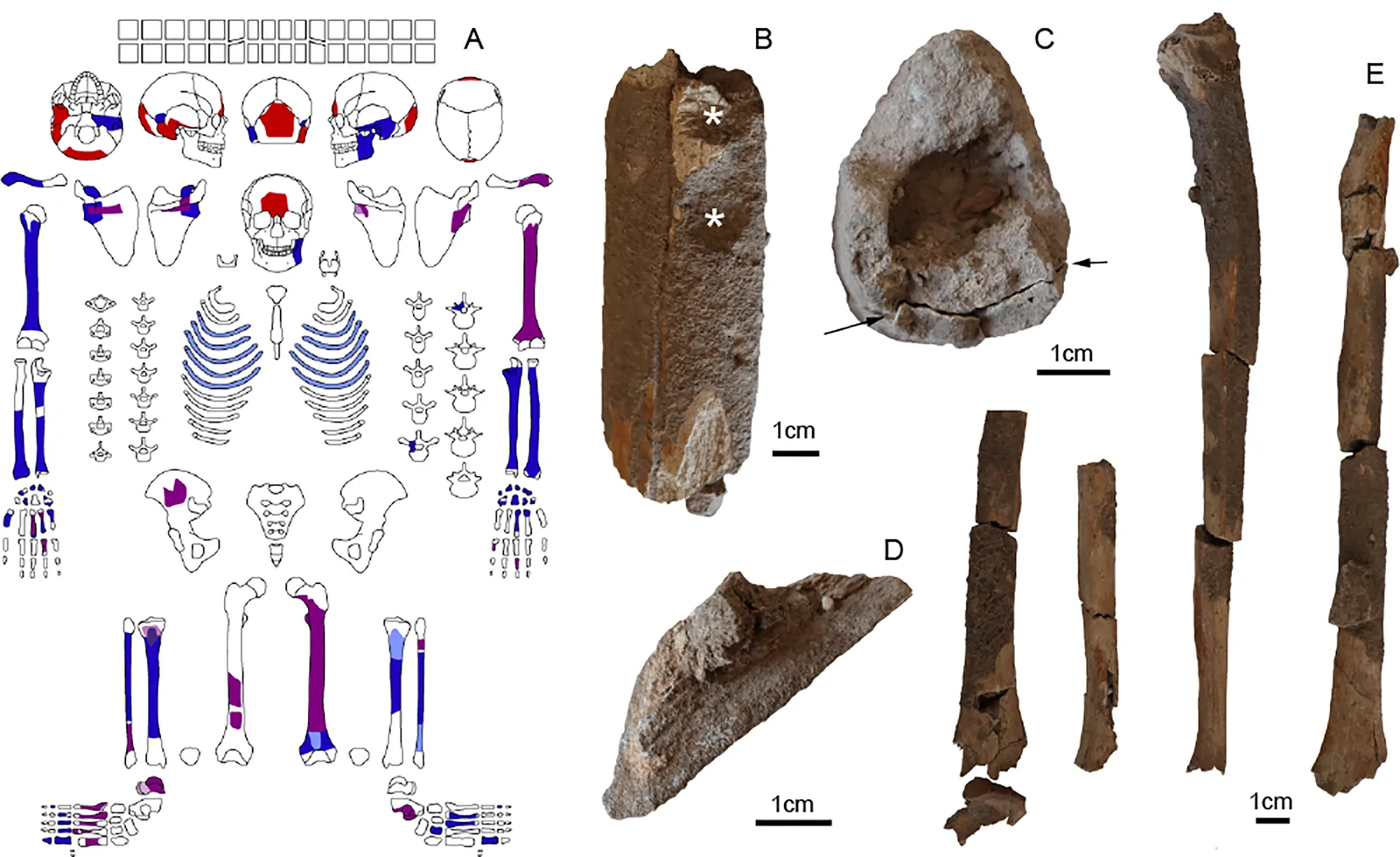
Individual from the burial in trench L09-65, showing taphonomic changes to the bones. (A) Representation of the skeleton of the young adult female. Bones marked in blue were excavated in 2022; the bones in red and violet were found during the first excavation in 2012. The red bones could belong to another (possibly male) individual; the violet and blue bones belong to the same individual. (B) Possible rodent gnawing marks on the left tibia in the shape of patches (stars). One is partly, and one is completely covered by calcareous deposits; (C) Broken shaft fragment of the right tibia, completely covered by calcareous deposits. A thick layer of compact bone on the dorsal surface peeled off (arrows), (D) Left tibia with double helical breaks; (E) Forearm bones covered by calcareous deposits. The shafts are fragmented, and the distal and proximal ends are missing. All images J. Gresky, DAI.

The burial in trench L09-65 was initially discovered in 2012. Due to time constraints, it was sealed at the time and only fully excavated in 2022. Several loci, including human bone fragments, were documented in the soil above the burial. Unfortunately, the boundary between the randomly distributed bone fragments from the room fill and the uppermost layer of the burial could not be clearly distinguished. This made it difficult to determine retrospectively which fragments originally belonged to the burial. Several bones and bone fragments could be attributed to the skeleton of a 20–30-year-old female, based on similarities in shape and size (Fig 4A). However, while the cranial bones found in 2012 suggest a male individual, the postcranial skeleton and the mandible fragment indicate a female. For this reason, the cranial fragments recovered in 2012 are marked in red (Fig 4A), whereas the fragments that most likely belong to the skeleton (blue) are shown in violet.

### Preservation and taphonomic changes

Except for the cranial bones, which cannot be assigned to the skeleton with certainty, all regions of the skeleton are represented by at least several bones (Fig 4A). The consistency of the bones is solid, and the surfaces are partly covered by a layer of calcareous deposits (Fig 4B–4E). Postmortem fragmentation of the bones is high: all of the epiphyses are broken and missing. The shafts of the long bones are broken mainly into three larger fragments, sometimes, particularly in the distal and proximal parts, into smaller pieces (Fig 4E). The edges of these fragments are all covered by a layer of calcareous deposits (Fig 4B–4D). Helical breaks [23,24] are present in both *tibiae* (Fig 4D). Possible rodent gnawing marks are present at the shafts of the right *ulna* and the left *tibia*. These are represented as patches with possible gnawing marks on the left *tibia* (Fig 4B) as well as opposing marks on both sides of the interosseous crest of the right *ulna.*

## Discussion

Since the beginning of excavations at Göbeklitepe in 1995, human bone fragments have been found scattered across the archaeological site. They appeared both in the deposits excavated from within the special buildings and in the soil between the architectural structures. Most fragments derive from the special buildings, likely a result of excavation bias, as these structures were the primary focus of fieldwork and therefore yielded the highest number of remains. The fragments were recovered from a matrix of limestone rubble mixed with stone artefacts and animal bones, and they generally lacked clear contextual associations. They occurred in small clusters, occasionally several in close proximity, though most were found as isolated pieces. Each fragment was recorded as an individual find, with assessments of age and sex where possible, along with descriptions of taphonomic and pathological alterations. However, the fragments could not be grouped or assigned reliably to specific individuals.

A long-standing discussion has surrounded the meaning of the scattered human bone fragments at Göbeklitepe, a question that could not previously be answered satisfactorily. With this paper, we are now able to address this issue in part, although several aspects will require further investigation in the future.

The central question was whether the human bone fragments were intentionally placed as part of a previously unknown burial practice, or had ended up in the fill of the special buildings by accident. To address this question, taphonomic alterations, which provide crucial information about the postmortem history of individuals, must be examined (e.g., 25).

In the case of the Göbeklitepe assemblage, a distinction can be made between natural and intentional causes. Natural causes include environmental factors such as climate, weathering, soil composition, and animal activity [25–29]. Weathering, rodent gnawing, and carnivore bite marks indicate that the bones were either exposed for some time or that there was open space within the burial or burrowing, making the body accessible for at least a certain period of time [29], while trampling and fragmentation could result from soil pressure or soil movement [12–15]. Intentional modifications, inflicted by humans [13,15,28,30–32], include cut marks, intentional breakage of the bones, and the deep incisions described above [11]. Perimortem breakage and heat alteration [12–15,33] may originate from either natural circumstances or deliberate human activity, and therefore require further detailed investigation.

The **first hypothesis** (deliberate deposition of selected human bones as part of secondary or tertiary burial practices) seemed plausible for several reasons. Cranial fragments belonging originally to three [11], now five individuals, were found to exhibit deep incisions along the midline of the cranium, indicating deliberate modification or treatment of particular individuals. This kind of skull treatment could have been reserved for important members of the group [8,11], but likewise for enemies. It appears that only a small group of individuals were treated in this manner, as most of the cranial fragments did not show such deep incisions [11]. The particular, so far singular, “skull cult” at Göbeklitepe, together with a clear over-representation of cranial elements in the assemblage (408 cranial vs. 283 postcranial fragments), may point to the intentional selection and placement of specific anatomical regions, particularly skulls. This interpretation corresponds well with earlier understandings of Göbeklitepe as a ritual centre, suggested by its monumental architecture and rich iconography. Within the latter, the depiction of headless human figures, most famously on Pillar 43 in Special Building D [34]; severed stone heads detached (intentionally?) from statues [35]; and numerous examples of statuary showing animals or birds grasping human heads, such as those from Nevali Çori [36] and Karahantepe [37], could indirectly suggest ritual or mortuary practices focused on the head.

At Göbeklitepe, this matches to finds of avifauna belonging to the corvid family, well known for their scavenging behaviour. Corvids are among the most prominent bird taxa at Göbeklitepe, occurring at frequencies four times higher than at other contemporaneous sites in the region. This unusual pattern could plausibly be linked to excarnation practices carried out in open areas [38–40]. Contemporaneous sites in the vicinity of Göbeklitepe present a diverse picture of this practice, which is partly comparable to Göbeklitepe. In most recent years, and in addition to the cranial fragments from Karahantepe mentioned above, newly initiated excavations at Sefertepe (EPPNB) have revealed spectacular burials of human remains; in one case, a skull was found buried within the floor plaster of a niche in the northwestern corner of a special building, while in a second case, at least twenty-one skulls were found in a small cell, now referred to as the “skull chamber”. The skulls had been placed on their bases, with almost all facing southeast [22,41]. The ”skull chamber” from Sefertepe shows notable parallels to the so-called *Skull Building* at Çayönü Tepesi, which in its later phases contained approximately 70 human skulls, while earlier phases also included complete skeletons [42,43]. Finally, at the PPNA/EPPNB site of Karahantepe, a new type of secondary burial practice has been identified, evidenced by the presence of multiple incisions and burn marks on crania [44].

A common pattern of the skull cult of these three sites is the localised arrangement of the skulls. In contrast, at Göbeklitepe, the bone fragments are scattered over the whole excavation area. This seems to be suspicious, as it might be expected that deliberate placements of bones would concentrate on particular areas instead of being dispersed in a rather random way over an area of 3000 square metres (main excavation area) as well as through all stratigraphic layers of the deposit (up to several metres in depth). Furthermore, skull fragments are mixed with postcranial fragments from individuals of both sexes and all age groups; this would argue against intentional selection.

The **second hypothesis** (burials from domestic contexts that became displaced and redeposited through erosion processes) is based on the domestic contexts of Göbeklitepe. The interpretation of Göbekli as a solely ritual centre [6], lacking significant permanent occupation, had to be revised in light of indisputable evidence of continuous settlement at Göbeklitepe throughout its entire sequence. The two burials presented here were discovered in spaces attributed to the EPPNB: the burial in trench DR1 lay beneath a plaster floor, while the burial in trench L09-65 was most likely situated below a floor as well, although this level is poorly preserved. In the latter case, the space lies on the western slope of the main excavation area, above and overlooking Special Building A. This building, together with Special Buildings B, C, and D, is situated in a depression and is flanked on three sides (west, north, and east) by accumulated tell deposits.

Burials beneath the floors of rooms are a well-documented practice throughout the PPNA and PPNB in Anatolia and the Levant [45–47], and their presence at Göbeklitepe was therefore expected. Helpful for the interpretation of the human bone fragments in the fill of the special buildings are the taphonomic changes of the skeletons in these burials:

The burial in trench L09-65 can be interpreted as a primary inhumation of a single individual. Although the burial appears largely undisturbed, the ends of the long bones are broken and missing, and the skeleton exhibits a highly fragmented pattern. This breakage may have resulted from soil pressure but could also be attributed to the effects of calcareous deposits. Over time, accumulated soil above the burial may have exerted pressure that fractured the larger bones, whereas smaller bones, having a reduced surface area, may have been less affected [48]. Calcareous deposits are the most destructive agents acting on bones at the site: they not only cover bone surfaces but also penetrate the bone matrix, causing cracking and, ultimately, fragmentation. While this process affects smaller bones as well, its impact is most pronounced on larger elements. The displacement of some smaller bones and bone fragments may be explained by animal activity. Although no burrows were observed during excavation, they could have been infilled by subsequent soil movement; the irregular, stony substrate also makes such features difficult to detect. The presence of rodent gnawing marks would further support this interpretation. This primary burial demonstrates that many of the observed alterations occurred within the burial context itself. The fragmentation of the bones, mainly postmortem transverse fractures, is therefore not the result of intentional shattering.

In the collective burial in trench DR1, none of the interred individuals was found in situ. Only at the lowest level, on the plaster floor upon which the bodies had originally been placed, were the distal parts of the left upper limb of one individual preserved in its anatomical position. All remaining bones had been rearranged and accumulated in three main clusters: in the eastern and western corners of the grave and in its central area. While no clear sorting of postcranial elements is apparent, the skulls had been collected in the eastern corner. Most skeletal regions are represented among the three individuals. The more fragile elements—such as ribs, vertebrae, and the pelvis—are largely absent, but this absence is best explained by taphonomic processes rather than deliberate removal. Conversely, the presence of small bones of the hands and feet indicates that complete bodies, or at least fully articulated limbs, were originally deposited. The absence of cut marks further suggests that the individuals were not interred as prepared or defleshed remains. A plausible explanation is that complete bodies were first placed in the grave and later moved aside to make room for subsequent interments. After the final burial, the bones appear to have been rearranged once more, although no further individuals were added and the grave was then closed. Given the relatively small size of the burial pit, the simultaneous placement of three bodies would not have been feasible. In this secondary arrangement, taphonomic alterations, particularly fragmentation, may be attributed to both the later movement of the bones within the grave and pressure from overlying sediments.

In summary, the taphonomic alterations observed in both burials, most notably the high degree of fragmentation, the extensive calcareous deposits, and the presence of rodent gnawing marks, closely correspond to the taphonomic characteristics of the human bone fragments recovered from the fill of the special buildings [11–15]. The single, clearly primary burial demonstrates that many of these changes had already occurred within the burial context itself. These observations support the hypothesis that at least part of the human remains found within the special buildings originated from subfloor burials located in buildings on higher-lying parts of the settlement mound, which were subsequently displaced by erosional processes (slope slides) and redeposited into the special buildings. Such landslide events would have resulted in an uneven distribution of bone fragments, thereby explaining the widespread occurrence of uncontextualised single human bone fragments within the fill matrix. While this offers a strong and plausible scenario, it does not exclude the possibility that additional mortuary practices – whether simultaneous or diachronic – were also taking place.

## Conclusion

By comparing the taphonomic features of the first excavated burials with those of the human bone fragments recovered from the fills of the special buildings at Göbeklitepe, a plausible explanation for the presence of the scattered fragments can now be offered for the first time. The dispersed and highly fragmented bones support the interpretation that they result from taphonomically induced processes. Specifically, the fragments appear to derive from subfloor burials situated in buildings on higher-lying parts of the settlement mound, which were subsequently displaced downslope through erosional events.

However, the identification of subfloor burials for the PPNB at Göbeklitepe does not preclude additional mortuary practices at the site, particularly given evidence from contemporaneous PPNB sites in the Taş Tepeler region, such as Sayburç and Sefertepe.

At Sayburç, dated to the 9th millennium BCE (EPPNB), human bones have been recovered from niches, and, in one case, scattered on and in front of a stone bench within a domestic dwelling [49]; in the latter case, the human remains, which consist primarily of crania and long bones belonging to a minimum of 19 individuals, some with evidence of burning, may have been removed from niches in the walls of the building before its abandonment [22,49].

The presence of comparable deposits at Göbeklitepe could explain the different taphonomic treatments of the human bones that cannot be attributed to primary subfloor interments. However, the deliberate deposition of selected human bones as part of secondary or tertiary burial practices seems to be less likely.

While the presence of primary and secondary subfloor burials might account for a large portion of the human bone fragments in the fill of the special buildings, we cannot rule out additional burial customs, such as the practice of a particular form of skull cult at Göbeklitepe [11]. Although it cannot yet be securely attributed to either the PPNA or PPNB, it finds clear parallels in the skull-related practices documented at Karahantepe (PPNA/EPPNB), Sefertepe (EPPNB), and Çayönü Tepesi, and later Çatalhöyük [50]. Further osteological analyses and absolute dating are essential to establishing a more precise chronological framework for the various forms of mortuary behaviour in Neolithic Anatolia, specifically at Göbeklitepe.

## Materials and methods

The position of all bones and bone fragments in the burials was recorded during the excavation process by photography, Structure-from-Motion, and drawings. In the lab, all bones and fragments were recorded in a database, and macroscopic photographs of each were taken. Taphonomic features, such as cut marks, impact of heat, rodent gnawing marks, and helical breaks, were recorded following the suggestion of [23,24,28,30–32]. For age estimation and sex determination, the methods suggested by Buikstra and Ubelaker [51] were applied. Due to the lack of many indicative features for age and sex determination, only some could be used. For sex determination, only features of the skull, mainly the mandible, could be recorded [52–54]. Age estimation in adults was based on the degree of dental wear [55,56], and the state of fusion of the cranial sutures [57,58], as well as the presence and severity of degenerative joint changes and osteoporosis. The state of epiphyseal closure of the long bones [59], long bone length [60,61] and dental development [62] were used to estimate the age of the adolescent. The detailed diagnostic criteria are described in the supporting information.

### Inclusivity in global research

Additional information regarding the ethical, cultural, and scientific considerations specific to inclusivity in global research is included in the Supporting Information (S2 File). Permissions for the study to take place were granted by the General Directorate of Cultural Assets and Museums, Ministry of Culture and Tourism of the Republic of Türkiye, the excavation director of Göbeklitepe, Necmi Karul, and the Şanlıurfa Museum, with its director Celal Uludağ. The human remains used in this study are currently stored at the Göbeklitepe excavation house.

No permits were required for the described study, which complied with all relevant regulations.

## Supporting information

S1 File Supplementary Text, Tables S1, S2. (DOCX)

S2 File Inclusivity-in-global-research-questionnaire. (DOCX)
