# Supplementary material for: First burials give insights into mortuary rites at Göbeklitepe, Early Neolithic Türkiye
Source: PLoS One. 2026 Sep 22;21(9):e0358979. doi: 10.1371/journal.pone.0358979 (PMC13596785; doi:10.1371/journal.pone.0358979)
Supplement: Supplementary file 1 [file pone.0358979.s001.docx]

Supplementary Materials for

**First burials give insights into mortuary rites at Göbeklitepe, Early Neolithic Türkiye.**

Julia Gresky and Lee Clare

*Corresponding author. Email: Julia.Gresky[@dainst.de](mailto:xxxxx@xxxx.xxx)

**This PDF file includes:**

Supplementary Text

Tables S1, S2

Supplementary Text

Additional information on the first burial in DR1-29

*Minimum number of individuals and representation of skeletal elements*

The cranium with the advanced fusion of the sutures was assigned to the female mandible and maxilla with severe antemortem tooth loss, representing the oldest individual. The cranial vault, with a very thick cross-section and open sutures, was assigned to the robust mandible of the young adult male. The third cranium of a female individual should fit the mandible of the adolescent; the frontal part with prominent frontal eminence and sharp orbital margins would fit the adolescent age group; however, the suture in C3 is already closed. The other parts of the cranial sutures seem to be open, although it is difficult to distinguish due to the calcareous deposits on the internal and external lamina. In summary, the cranium will be assigned to the adolescent in the following text.

There are two additional bones, metatarsal shafts and heads, which could not be assigned to any of the individuals by their specific, roundish shafts. By their number, they could fit the two older individuals, but by their shape, they might represent a fourth individual. Only the sternum is missing in all individuals; in the two adults, the bones of the spine and ribs are missing.

*Preservation*

The surfaces are partly covered by a layer of calcareous deposits. This layer is generally thin; however, on the internal lamina of all three crania, it appears as a thick layer, with inclusions of grit and smaller bone fragments. The surface of the majority of the bone fragments (61.4%, n=116/189) is covered between 70-100%. Covering of 30-60% of the surface is present in 25.9% (n= 49/189) of fragments, and a slight covering between 1-20% of the surface can be found in 12.7% (n=24/189) of cases.

*Fragmentation*

There are no spiral fractures, but several helical breaks (Tab. S1). Two occurred in individual 2 at the right *humerus,* cutting it horizontally (Fig. 2E,F), and one horizontal fracture occurred on the proximal shaft of the fifth right metacarpal. Two are in individual 3 running slightly oblique through the shaft of the third proximal phalanx of the left hand and the right rib, cutting the shaft vertically (Fig. 2H).

*Rodent gnawing marks*

Most of the bones with possible evidence of rodent gnawing marks belong to individual 2 (Tab. S2). Clear gnawing marks are present at the distal rims of the shaft of the right *humerus* (Fig. 2F,G). Other marks are more localized in groups on the external lamina of the right side of the occipital bone, the right parietal bone, and the right scapula. These depressions are covered by calcareous deposits and therefore cannot be verified. The only sign of a rodent gnawing mark on another skeleton is in individual 3, where one of the ribs shows parallel scratches (Fig. 2I). However, as they are of a different colour to the surrounding bone, they might have resulted from more recent activity.

*Age estimation and sex determination*

Individual 1 (Fig. 2A): 35+ years, female

Antemortem tooth loss (fully closed alveoli of teeth 16, 15, 36, and partly closed of 36, 42) indicates an age older than 30 years. Dental wear of the only present tooth 47 (grade 4 (38)) is concordant with an age of 25-35 years. However, the possible missing antagonists might also explain the less severe wear. Closure of the cranial sutures (C fully closed, S1/2 fully closed, S3/4 not determinable, L not closed) points to an age older than 30 years. There are no severe degenerative changes of the joints (*scapula, radius, ulna*, hip, hand, and feet). The compact bone of the *femur* and *humerus* is very porous, particularly in the area of the *linea aspera*.

For sex determination, the mandible could be used. The prominent mental tubercle on the mental trigone points to the female sex, as does the gracility of the mandible and the whole skeleton.

Individual 2 (Fig. 2B): 20-30 years, male

Dental wear points to an age of 17-25 years. All epiphyseal lines of the present long bones (*radius, ulna, femur*, bones of hands and feet) are closed, indicating an age older than 17-22 years. The cranial sutures (S2-4 and L) are not closed, suggesting an age of less than 30 years. The joint surfaces show no signs of wear.

The mandible suggests male sex (height of the body; the lower rim of the mandibular body is roundish and thick, although the mental tubercle on the mental trigone is rather prominent). The right mastoid process is indistinct.

Individual 3 (Fig. 2C): 11-14 years, female

The state of epiphyseal closure of the long bones (*humerus, radius, femur, tibia*, hands, and feet) and the *scapula* and *pelvis* suggests an age younger than 14 years. An approximate measurement was taken from both *humeri,* suggesting an age of 12-13 (14) years. Dental development is concordant with an age of 11-14 years (second molar fully erupted, but the tips of the roots are not yet completely fused; length of the roots of tooth 44).

For sex determination, the mandible was present: The mental tubercle is slightly prominent, but it is thick, so it is not decisive for one sex. The gonial angle does not show lateral flaring, indicating a rather female sex. The body height is between the two other individuals.

Considering the cranium as belonging to this skeleton, a female sex can be assigned (prominent frontal eminence, sharp orbital margin).

Additional information on the second burial in L 09-65

*Minimum number of individuals*

The burial in trench L09-65 was initially discovered in 2012. Due to time constraints, it was sealed and finally excavated in 2022. Several loci, including human bone fragments, were documented in the soil on top of the burial. Unfortunately, the border between the randomly distributed bone fragments of the room-fill and the first layer of the burial could not be separated. This situation made it difficult to decide retrospectively which bone fragments originally belonged to the burial. Several bones and bone fragments could be attributed to the skeleton because of their similar shape and size (Fig. 4A). However, while the cranial bones point to a male individual, the entire postcranium and the mandible fragment indicate a female individual.

*Preservation and representation of skeletal elements*

Except for the cranial bones, which cannot be assigned to the skeleton definitively, all regions of the skeleton are represented by at least some bones.

The consistency of the bones is solid, and the surfaces are partly covered by a layer of calcareous deposits (Fig. 4). In 30.2% (n=13/43), the surface is covered between 70-100%. Covering of 30-60% of the surface is present in 60.5% (n=26/43). Slight covering between 1-20% of the surface can be found in 9.3% (n=4/43). The cover is mainly thin; on some bones, like the *tibia*, it is quite thick (Fig. 4B-D). In the *humerus* and *tibia*, fragments of the surface (calcareous deposit and attached bone surface) peeled off shell-like when the bones were washed (Fig 4C).

*Fragmentation*

The bones are fragmented; all of the epiphyses are broken and missing; the shafts are broken mainly into three bigger fragments, sometimes, particularly in the distal and proximal parts, into smaller pieces (Fig. 4E). The endings of these fragments are all covered by a layer of calcareous deposits.

Helical breaks are present in both *tibiae*. The left *tibia* shows a double helical break of the dorsal shaft slightly proximal to the middle of the shaft (Fig. 4B,D). The broken edges and the complete shaft are covered by a layer of calcareous deposits. The right *tibia* shows two helical breaks distal to the middle of the shaft. One is on the lateral-dorsal surface, and the fragment chipped off like a flake. The shaft distal to it also broke in a helical break. The surfaces are covered by calcareous deposits, as is the complete shaft.

*Rodent gnawing marks*

Rodent gnawing marks were present on the shafts of the right *ulna* and the left *tibia.* These are represented as patches with gnawing marks on the left *tibia* (Fig. 4B) as well as opposing marks on both sides of a crest at the right *ulna*.

*Age estimation and sex determination*

The epiphyses of the long bones are all missing, but the completely closed epiphyseal lines of the phalanges of the hand and feet, as well as the distal left *femur* and right r*adius*, indicate an adult age. There are very slight degenerative changes, suggesting a young adult age between 20-30 years.

The *pelvis* and most parts of the skull are missing. A fragment of the left mandible and the left mastoid process are not distinctive for either male or female sex (the mastoid process has a broad base; the tip is broken postmortem; the dimension of the gonial angle rather points to a female (ca. 140°); it is slightly everted due to a robust attachment of the masseter muscle indicating a rather male sex). Because the bones are very small and gracile, a female sex might be more likely (especially compared with the very robust bone fragments).

The cranial bones, which were found close to the skeleton and therefore could belong to it, are of rather male sex (The glabella region and the supraorbital ridge are pronounced).

Table S1.

Breakage pattern: Smooth fracture margins in bones of individuals 2 and 3 from the burial in trench DR1-29.

| **Individual** | **Location** | **Signature** | **Bone** | **Fragment Detail** | **Fracture** |
| --- | --- | --- | --- | --- | --- |
| Ind. 2 |  | GT17_HB_1854 | Right *humerus* | *Humerus* is comprised of two fragments | Horizontally through the shaft |
| Ind. 2 | south | GT17_HB_1855 | Right 5^th^ metacarpal | Proximal shaft | Horizontally through the shaft |
| Ind. 3 | north | GT17_HB_1854 | Right rib | Rib comprises four fragments | Vertically through the shaft |
| Ind. 3 | south | GT17_HB_1855 | 3^rd^ proximal phalanx of the left hand | Proximal shaft | Slightly oblique through the shaft |

Table S2.

Rodent gnawing marks on the bones of individuals 2 and 3 from the burial in trench DR1-29.

| **Individual** | **Location** | **Signature** | **Bone** | **Fragment Detail** | **Gnawing marks** |
| --- | --- | --- | --- | --- | --- |
| Ind. 2 | lower N skull | GT17_HB_1924 | Occipital bone, right side | Fragment with transverse sulcus | Possible, covered by deposits |
| Ind. 2 | lower N skull | GT17_HB_1924 | Parietal bone right |  | Possible, covered by deposits |
| Ind. 2 | North | GT17_HB_1854 | *Scapula* right | Two fragments | Possible, covered by deposits |
| Ind. 2 | bones in the remaining soil in south part |  | *Humerus* right | Distal third of the shaft | Lateral supracondylar ridge |
| Ind. 3 | North | GT17_HB_1854 | Rib right | Rib comprises four fragments | Possible, but rather recent activity |
